# Acetylcholinesterase-Inhibiting Activity of Pyrrole Derivatives from a Novel Marine Gliding Bacterium, *Rapidithrix thailandica*

**DOI:** 10.3390/md20080029

**Published:** 2008-10-13

**Authors:** Yutthapong Sangnoi, Oraphan Sakulkeo, Supreeya Yuenyongsawad, Akkharawit Kanjana-opas, Kornkanok Ingkaninan, Anuchit Plubrukarn, Khanit Suwanborirux

**Affiliations:** 1 Department of Industrial Biotechnology, Faculty of Agro-Industry, Prince of Songkla University, Hat-Yai, Songkla 90112, Thailand; 2 Marine Natural Products Research Unit, Department of Pharmacognosy and Pharmaceutical Botany, Faculty of Pharmaceutical Sciences, Prince of Songkla University, Hat-Yai, Songkhla 90112, Thailand; 3 Department of Pharmaceutical Chemistry and Pharmacognosy, Faculty of Pharmaceutical Sciences, Naresuan University, Phitsanulok 65000, Thailand; 4 Center for Bioactive Natural Products from Marine Organisms and Endophytic Fungi (BNPME), Department of Pharmacognosy, Faculty of Pharmaceutical Sciences, Chulalongkorn University, Patumwan, Bangkok 10330, Thailand

**Keywords:** pyrroloquinolines, phenylpyrroles, gliding bacteria, *Rapidithrix thailandica*, acetylcholinesterase inhibitors

## Abstract

Acetylcholinesterase-inhibiting activity of marinoquinoline A (**1**), a new pyrroloquinoline from a novel species of a marine gliding bacterium *Rapidithrix thailandica*, was assessed (IC_50_ 4.9 *μ*M). Two related pyrrole derivatives, 3-(2′-aminophenyl)-pyrrole (**3**) and 2,2-dimethyl-pyrrolo-1,2-dihydroquinoline (**4**), were also isolated from two other strains of *R. thailandica*. The isolation of **3** from a natural source is reported here for the first time. Compound **4** was proposed to be an isolation artifact derived from **3**. The two isolated compounds were virtually inactive in the acetylcholinesterase-inhibitory assay (enzyme inhibition < 30% at 0.1 g L^−1^).

## 1. Introduction

One of the most important roles of acetylcholine in the brain is to govern the connectivity among neurons, thereby regulating the brain’s cognitive functions. A deficit in acetylcholine, especially in the basal forebrains, is a neurochemical characteristic of patients clinically diagnosed with Alzheimer’s disease (AD). Using acetylcholinesterase (AChE) inhibitors to retard the catabolic hydrolysis of acetylcholine, therefore compensating such deficiency particularly at the synaptic terminals, has been suggested as one of the most direct remedies for AD treatment. To date, only three AChE inhibitors; donepezil, rivastigmine, and galantamine, have been approved by US FDA for the treatment of AD. Whereas it is arguable whether such drugs provide a long-term treatment or are merely a symptom intervention, they are one of only a few effective approaches available for the treatment of AD [1,2].

Recently, we reported the isolation and structure elucidation of marinoquinoline A (**1**), a new alkaloid possessing an unprecedented pyrrolo[2,3*-c*]quinoline skeleton from a novel marine gliding bacterium *Rapidithrix thailandica* [3,4]. The structural similarity between **1** and tacrine (**2**), a potent AChE inhibitor, prompted us to subject the compound to the AChE inhibiting assay, as well as to search for additional pyrrole/pyrroloquinoline derivatives from the bacterium. Here, the isolation and structure determination of the phenylpyrrole analogs of **1** will be reported, along with the AChE-inhibiting activity of **1** and related compounds.

## 2. Results and Discussion

Marinoquinoline A (**1**) was obtained from the gliding bacterium *R. thailandica* TISTR 1742 as reported earlier [3]. Given the close resemblance between **1** and **2**, we speculated that **1** might bind to the enzyme AChE in a manner similar to that of **2**. Consequently, **1** was assayed against AChE using *Torpedo californica* AChE. As expected, the compound exhibited a strong inhibition with an IC_50_ of 4.9±0.9 *μ*M (referred to standard galantamine, IC_50_ 0.6±0.1 *μ*M) with no appreciable cytotoxicity against the panel of cancer cell lines (> 80% cell viability at 20 *μ*M).

**Figure:**
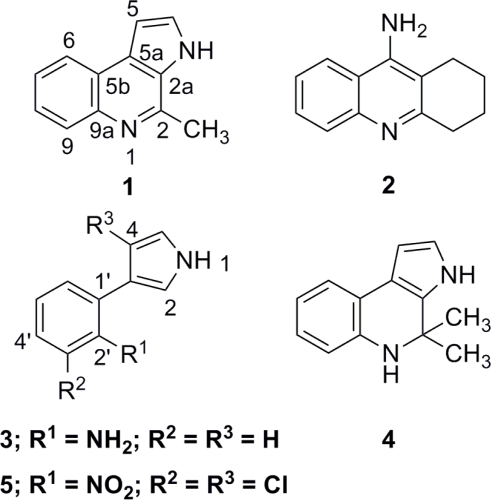


Based on the promising results, two other strains of *R. thailandica* were selected for the chemical investigation in an effort to discover related pyrrole/pyrroloquinoline derivatives. The gliding bacterial strains TISTR 1749 and SH5.13.2 were separately isolated from a submerged sea grass blade collected from Fossil Shell Beach Park, Krabi Province, and from debris collected from Koh-Kham, Chonburi Province, respectively. The 16S rDNA analysis of the bacterial isolates suggested that they were identical to each other. The BLAST search with the 16S rDNA gene sequences in GenBank database indicated that both strains belonged to the gliding bacterium *R. thailandica*, gen. nov., sp. nov. (family Flammeovirgaceae), with 99% similarity in gene sequences to the type strain TISTR 1750^T^ [4].

The large scale fermentations of strains TISTR 1749 and SH5.13.2 were carried out in skim milk and CY media, respectively. Amberlite XAD-16, added to the fermentation broths of each strain, was harvested and eluted with MeOH, and each extract was subjected to the further chromatographic separation. Extract from the strain TISTR 1749 yielded **3** and **4**, whereas that of SH5.13.2 yielded **3** as the main product.

Compound **3** possesses a molecular formula of C_10_H_10_N_2_ according to the molecular ion signal at *m/z* 158 observable in the EI mass spectrum. This was confirmed by the high resolution mass of 158.0852 (EI; calc for C_10_H_10_N_2_ 158.0842). The ^1^H NMR spectrum (500 MHz, DMSO-*d*_6_, Table 1) showed nine resonances, seven of which belonged to two aromatic spin systems, while the other two were exchangeable proton signals. The first aromatic spin system, resonating at *δ* 6.55 (ddd, *J* = 7.5, 7.3, 1.3 Hz; H-5′), 6.69 (dd, *J* = 7.9, 1.3 Hz; H-3′), 6.88 (ddd, *J* = 7.9, 7.3, 1.3 Hz; H-4′), and 7.10 (dd, *J* = 7.5, 1.5 Hz; H-6′), was characteristic to a 1,2-disubstituted benzene ring. Another spin system was elucidated as 3-substituted pyrrole moiety according to the characteristic proton signals at *δ* 6.25 (ddd, *J* = 2.5, 2.3, < 1 Hz; H-4), 6.83 (ddd, *J* = 2.5, 2.3, 2.3 Hz; H-5), and 6.96 (ddd, *J* = 2.3, 2.3, < 1Hz; H-2). This agreed well with the observation of the low-fielded exchangeable proton at *δ* 10.90 (br s), typically assigned to H-1 of pyrrole. The connection of the two aromatic rings was achieved via the HMBC long-range correlation from C-3 to H-6′. The presence of a primary amino group was deduced from the characteristic IR absorption band at *ν*_max_ 3400 cm^−1^, and from the exchangeable proton resonance at *δ* 4.70 (br s, 2H; 2′-NH_2_). Attachment of the amino group onto C-2′ (*δ* 144.8) of the phenyl ring furnished the structure of **3** as 3-(2′-aminophenyl)-pyrrole. The nOe between each pair of protons along the circumference of the structure, i.e., H-1 ↔ H-2 ↔ 2′-NH_2_ ↔ H-3′ ↔ H-4′ ↔ H-5′ ↔ H-6′ ↔ H-4 ↔ H-5 ↔ H-1, strongly supported the proposed structure. The structure of **3** has been proposed as one of theoretical biosynthetic intermediates of the antibiotic pyrronitrin (**5**) [5–7], previously reported from several pseudomonads and related bacteria [5,8–10]. This hypothesis was challenged in a recent report by Kirner et al [11], who suggested that **3** was in fact either a side product or biosynthetic dead-end of **5**. In either case, however, **3** has never been actually isolated from any natural sources.

The molecular formula of **4** was proposed to be C_13_H_14_N_2_ as deduced from the high resolution mass at *m/z* 198.1122 in the HR-EI mass spectrum (calc for C_13_H_14_N_2_ 198.1154). From the ^1^H NMR spectrum (500 MHz, DMSO-*d*_6_, Table 2), seven proton resonances characteristic of a 1,2-disubstituted benzene (*δ* 6.45, ddd, *J* = 7.7, 7.5, 1.0 Hz, H-7; 6.50, dd, *J* = 7.9, 1.0 Hz, H-9; 6.73, ddd, *J* = 7.9, 7.7, 1.3 Hz, H-8; and 7.08, dd, *J* = 7.5, 1.3 Hz, H-6) and of a 2,3-disubstituted pyrrole (*δ* 6.20, dd, *J* = 2.6, 2.4 Hz, H-5; 6.64, dd, *J* = 2.8, 2.6 Hz, H-4; and 10.74, br s, H-1) suggested that **3** and **4** were closely related. The 40 mass units higher than that of **3**, and the additional NMR signals, which include those of a quarternary carbon at *δ* 52.1 (C-2) and two geminal methyl at *δ*_H_ 1.42 (s, 6H; 2-CH_3_) and *δ*_C_ 30.6 (2C), indicated that **4** was a dimethyl dihydroquinoline analog of **3**. The structure of **4** was therefore proposed as 2,2-dimethyl-pyrrolo-1,2-dihydroquinoline. Also, similar to **3**, a network of nOes along the circumference of the structure was observed, which strongly supported the proposed structure of **4**.

Whereas the fermentation broth of strain TIRTR 1749 led to the isolation of two major components, that of SH5.13.2 primarily yielded only **3**. The difference in the chemical compositions between the two strains raised the questions whether **4** was genuinely produced by *R. thailandica* TISTR 1749 or whether the compound was in fact an isolation artifact. Careful examination of the ^1^H NMR spectra of the MeOH extract and subsequent fractions showed that only after the first chromatographic step, in which acetone was extensively used, were all the resonances related to **4**, especially the prominent methyl signal, observable. This suggested that **4** was potentially an artifact, presumably generated from SiO_2_-catalyzed Pictet-Spengler reaction between **3** and acetone. This was also supported by the absence of **4** in the extract from strain SH5.13.2, where acetone was avoided.

The AChE inhibitory activity and cytotoxicity of **3** and **4** were assessed. Surprisingly, both were inactive in the AChE inhibition and in the cytotoxicity bioassays (enzyme inhibition < 30% at 0.1 g L^−1^; > 80% cell viability at 20 g L^−1^, respectively). Despite sharing a related skeleton, the three compounds exhibited different activities. It is proposed here that the binding behavior to AChE of the pyrroloquinolines may relate to the extended aromaticity of the quinoline moiety of **2**, which may have *π*, *π*-interaction in the gorge of AChE [12,13].

In summary, we have demonstrated the potential of **1** as a new and potent AChE inhibitor. The investigation of two other strains of the novel marine gliding bacterium *R. thailandica* also led to the isolation of pyrrole derivatives, **3** and **4**. Although **4** was suggested here as a possible isolation artifact generated from the condensation between **3** and acetone, the structures of the two isolated compounds have nonetheless never been reported from any natural and wild-type bioresources. In addition, without any intermediates related to **5** observable in the extract, the isolation of **3** as a major component from the marine gliding bacterium confirms the previous conclusion contributed in [11] that, unless produced from otherwise entirely different biosynthetic pathway, **3** (and presumably **1**) can be presumed as a biosynthetic dead-end branched from that of **5**, which are commonly found in several terrestrial bacteria.

## 3. Experimental

### 3.1. General

Unless stated otherwise, all chemicals, chromatographic solvents, and media were used as purchased. Sea water for all media preparation was filtered through cotton plug before used. The classical chromatographic separations were all performed using SiO_2_ (Scharlau^®^, 230–400 mesh), whereas the size-exclusion one was operated on a Sephadex LH20 (GE Helathcare^®^) column. Preparative HPLC was performed on a Water^®^ 600E multisolvent delivery system, which was connected to a Water^®^ 484 UV detector, and a Rheodyne^®^ 7125 injector port. IR spectra were obtained from a Jasco IR-810 infrared spectrometer. UV spectra were measured on a Spectronic Genesys 5 spectrometer. Mass spectra were operated on a Micromass LCT mass spectrometer. The NMR experiments were performed on a 500-MHz FT-NMR Varian Unity Inova 500 spectrometer, and were all referenced to the solvent signals (DMSO-*d*_6_; *δ* 2.49 of trace C_2_D_5_HSO for ^1^H; *δ* 39.7 for ^13^C) as the internal standard.

### 3.2. Bacterial isolation and purification

The gliding bacteria TISTR 1749 and SH5.13.2 were isolated from a submerged sea grass blade collected from Fossil Shell Beach Park, Krabi Province, and from debris collected from Koh-Kham, Chonburi Province, respectively. For each isolate, a small piece of the collected specimen was cut and allowed to stand at an ambient temperature on a Petri dish of either SWG medium (for TISTR 1749) containing (g L^−1^ of sea water) monosodium *L*-glutamate (1), NH_4_NO_3_ (0.01), K_2_HPO_4_ (0.01), agar (15) [14]; or a crystal violet-supplemented (1 g L^−1^) SWG medium (for SH5.13.2) [modified from 15]. Once swarm colonies were observed, a cut piece of agar medium bearing one clean edge of each colony was transferred to a new subculture plate of SAP_2_ solid medium, containing (g L^−1^ of sea water) tryptone (1) and agar (15) [14, modified from 16]. The pure culture of the strain TISTR 1749 was obtained by means of micromanipulation technique [3,14], whereas that of SH5.13.2 was via repeated subculturing on SAP_2_ medium at ambient temperature. The reference culture of the strain TISTR 1749 was lodged at the culture collection of Thailand Institute of Scientific and Technological Research, Pathum-Thani, Thailand, and that of SH5.13.2 was at the Marine Biotechnology Laboratory, Department of Industrial Biotechnology, Faculty of Agro-Industry, Prince of Songkla University, Songkhla, Thailand.

### 3.3. Taxonomic identification

The taxonomic identification of both strains was based on the 16S rDNA analysis, performed as described in [3,14]. In brief, the 16S rDNA was amplified by PCR technique using the 16S rDNA universal primers BF1 and BR1. The purification of the PCR products was carried out using GFX PCR DNA and gel band purification kit (Amersham®). Sequencing reaction was performed using the ABI PRISM BigDye Terminator cycle sequencing kit (Applied Biosystem®). Sequencing was edited and assembled using the BioEdit program [17] and partial 16S rDNA sequences were compared with those available in the DNA databases using the BLASTN algorithm for close evolutionary relatives. The complete 16S rDNA sequences determined for representative strains of each group were aligned with the sequences of reference organisms derived from the database using the ClustalX 1.8.

### 3.4. Large scale fermentation

The medium for pre-culture and large-scale production of each strain of the gliding bacteria was as followed; SAP_2_ liquid medium (g L^−1^ of sea water) containing tryptone (1), yeast extract (1) [14, modified from 16]; CY medium (g L^−1^ of sea water) containing casitone (10), malt extract (2), yeast extract (1) [modified from 18]; skim milk medium (g L^−1^ of sea water) containing skim milk powder (5), yeast extract (3) [modified from 19].

The pre-culture of each bacterial strain was performed separately in 250-mL flasks each containing 30 mL of SAP_2_ liquid medium (72 h, 25°C, 200 rev min^−1^). A 5-mL portion of the pre-culture broth from each strain was then separately transferred into a 250-mL flask (×80) of 100-mL production medium as stated accordingly. An *in promptu* extraction was carried out using amberlite XAD-16 resins (2 g per 100 mL medium), added prior to sterilizing of each aforementioned production medium. After a 7-day incubation (25°C, 200 rev min^−1^), the resin beads from each strain were separately harvested via nylon mesh, rinsed with water, and extracted with MeOH (2×100 mL).

### 3.5. Extraction and compound isolation

The isolation of **1** was as per our previous report. The identification was performed on the basis of spectral analysis, from which all impurities were undetectable.

The MeOH extract from the fermentation broth of the strain TISTR 1749 was subjected to a solvent partitioning protocol to yield hexane-, CH_2_Cl_2_-, and *n*-BuOH-extracts. The CH_2_Cl_2_-extract, which showed prominent ^1^H NMR signals for phenylpyrroles, was selected for the further isolation.  The chromatography of the extract on a SiO_2_ column (step gradient solvents, ramping from 20 to 30% acetone in hexane, then 5 to 50% MeOH in CH_2_Cl_2_), then on a SiO_2_ HPLC column (250×7 mm Econosil®, 10 *μ*m; continuous gradient 5 to 35% *i*-PrOH in hexane in 15 min, 2 mL min^−1^; 254 nm) yielded **3** (12 mg; *t*_R_ 19 min) and **4** (27 mg; *t*_R_ 13 min).

The MeOH extract from the broth of SH5.13.2 was directly chromatographed over Sephadex LH20 (MeOH), followed by RP-C18 HPLC (250×10 mm Phenomenax®, 10 *μ*m; 70% aq MeOH, 5 mL min^−1^; 210 nm), and **3** (35 mg; *t*_R_ 31 min) was obtained.

**3-(2′-Aminophenyl)-pyrrole (3).** Brownish orange glass; UV (MeOH) *λ*_max_ (log *ɛ*) 212 (4.07), 302 (3.34) nm; IR (thin film) *ν*_max_ 3400, 2910, 1610 cm^−1^; EIMS *m/z* (% relative intensity) 158 [M^+^] (100), 130 (85), 84 (52), 66 (65); HR-EIMS *m/z* 158.0853 (calc for C_10_H_10_N_2_ 158.0842); ^1^H and ^13^C NMR (DMSO-*d*_6_; 500 MHz for ^1^H) see Table 1.

**2,2-Dimethyl-pyrrolo-1,2-dihydroquinoline (4).** Purplish blue glass; UV (MeOH) *λ*_max_ (log *ɛ*) 230 (4.41), 322 (3.67) nm; IR (thin film) *ν*_max_ 3400, 2905, 1520, 1450, 1400 cm^−1^; EIMS *m/z* (% relative intensity) 198 [M^+^] (15), 183 (100); HR-EIMS *m/z* 198.1122 (calc for C_13_H_14_N_2_ 198.1154); ^1^H and ^13^C NMR (DMSO-*d*_6_; 500 MHz for ^1^H) see Table 2.

### 3.6. Bioactivity determination

The AChE inhibitory assay was conducted (in triplicate) according to the protocol described by Ingkaninan et al [20, modified from 21]. To a solution of 125 *μ*L 5,5′-dithiobis[2-nitrobenzoic acid] (3 mM), 25 *μ*L acetylthiocholine iodide (1.5 mM), 50 *μ*L Tris-HCl buffer (pH 8.0; 5.0 mM), and 25 *μ*L of each tested compound in Tris-HCl buffer was added 25 *μ*L *Torpedo californica* AChE (type VI-S, EC 3.1.1.7; 0.28 U mL^−1^; Sigma^®^). The developing yellow color was measured at 405 nm over 2 min with a 5-s interval using a CERES UV900C microplate reader (Bio-Tek Instrument). The resulting velocity was calculated and used for the determination of the enzyme activity. The IC_50_ of each compound was calculated using Prism^®^ software (Graph Pad Inc.), and was referred to the potency of standard galantamine (IC_50_ 0.6±0.1 *μ*M).

Cytotoxicity determination was performed according to the sulphorrhodamine B assay protocol described in [22]. The paneled cancer cell lines used here include MCF-7 (human breast adenocarcinoma), HeLa (human cervical carcinoma), KB (human oral epidermoid carcinoma), and HT-29 (colorectal carcinoma). The potency was referred to camptothecin as a standard reference (IC_50_s 0.6–6 × 10^−6^ *μ*M).

## Figures and Tables

**Table 1.** NMR spectral data of **3** (DMSO-*d*_6_; 500 MHz for ^1^H).

position	^1^H^a^ (mult.; *J* in Hz)	^13^C (mult.)
1	10.90 (br s)	-
2	6.96 (ddd; 2.3,2.3,<1)	115.8 (CH)
3	-	121.1 (C)
4	6.25 (ddd; 2.5,2.3,<1)	107.3 (CH)
5	6.83 (ddd; 2.5,2.3,2.3)	118.3 (CH)
1′	-	121.6 (C)
2′	-	144.8 (C)
3′	6.69 (dd; 7.9,1.3)	115.2 (CH)
4′	6.88 (ddd; 7.9,7.3,1.5)	126.2 (CH)
5′	6.55 (ddd; 7.5,7.3,1.3)	116.9 (CH)
6′	7.10 (dd; 7.5,1.5)	128.9 (CH)
2′-NH_2_	4.20 (br s; 2H)	-

a Note: Unless stated otherwise, each proton signal integrated as 1 H.

**Table 2.** NMR spectral data of **4** (DMSO-*d*_6_; 500 MHz for ^1^H).

position	^1^H^a^ (mult.; *J* in Hz)	^13^C^b^ (mult.)
1	5.70 (br s)	-
2	-	52.1 (C)
2a	-	132.2 (C)
3	10.74 (br s)	-
4	6.64 (dd; 2.8,2.6)	117.8 (CH)
5	6.20 (dd; 2.6,2.4)	101.5 (CH)
5a	-	113.5 (C)
5b	-	119.1 (C)
6	7.08 (dd; 7.5,1.3)	121.0 (CH)
7	6.45 (ddd; 7.7,7.5,1.0)	116.2 (CH)
8	6.73 (ddd; 7.9,7.7,1.3)	125.0 (CH)
9	6.50 (dd; 7.9,1.0)	112.7 (CH)
9a	-	141.7 (C)
2-CH_3_	1.42 (s; 6H)	30.6 (CH_3_, 2C)

a Note: Unless stated otherwise, each proton signal integrated as 1 H.

b Unless stated otherwise, each carbon signal is equivalent to 1 C.
